# Editorial: Improving the delivery of pre-exposure prophylaxis (PrEP) to eliminate vertical HIV transmission

**DOI:** 10.3389/frph.2024.1382548

**Published:** 2024-04-16

**Authors:** Irene Njuguna, Friday Saidi, Dvora Joseph Davey, Benjamin H. Chi, Jillian Pintye

**Affiliations:** ^1^ Department of Medical Research, Kenyatta National Hospital, Nairobi, Kenya; ^2^ Department of Global Health, University of Washington, Seattle, WA, United States; ^3^ UNC Project Malawi, Lilongwe, Malawi; ^4^ Department of Obstetrics and Gynecology, University of North Carolina at Chapel Hill, Chapel Hill, NC, United States; ^5^ Department of Obstetrics and Gynecology, Kamuzu University of Health Sciences, Lilongwe, Malawi; ^6^ Division of Infectious Diseases, David Geffen School of Medicine, University of California, Los Angeles, CA, United States; ^7^ Department of Epidemiology and Biostatistics, University of Cape Town, Cape Town, South Africa

**Keywords:** pre-exposure prophylaxis (PrEP), HIV, pregnancy and lactation, prevention of vertical transmission of HIV, PrEP implementation

**Editorial on the Research Topic** Improving the delivery of pre-exposure prophylaxis (PrEP) to eliminate vertical HIV transmission

HIV pre-exposure prophylaxis (PrEP) significantly reduces new HIV infections (1). Among pregnant and lactating cisgender women in high HIV prevalence settings PrEP offers dual benefits for maternal and infant HIV prevention and is increasingly integral to vertical transmission prevention programs (2, 3). Many countries in East and Southern Africa with high HIV burden have integrated oral PrEP into HIV prevention programs (4), in the form of daily oral tenofovir disoproxil fumarate (TDF) containing regimens. While daily oral TDF-based PrEP use in pregnancy and lactation is considered safe and effective (5), only recently are data on PrEP implementation and extended safety emerging (6–8). As additional PrEP options become available (9), there is a need for more evidence on how to ensure effective antenatal and postnatal use (10).

Because of its high relevance to public health—and to global goals to eliminate pediatric HIV—we sought to highlight new research in this field. The result is this collection, which includes 13 articles of work done in sub-Saharan Africa (South Africa, Kenya, Eswatini, Zambia, Malawi, Lesotho, and Uganda) and the United States, demonstrating the importance of the topic globally. This body of work followed four major themes: (1) client knowledge, attitudes, and beliefs about PrEP (2) the PrEP care continuum, (3) healthcare provider experiences and attitudes and (4) PrEP safety, effectiveness, and delivery in pregnancy (Table 1). This work spanned the periconceptional period, pregnancy, and lactation. From this collection, we take away important lessons that will assist in advancing the field of PrEP provision of pregnant and lactating people.

**Table 1:** Summary of studies on PrEP in pregnant and lactating women in this collection.

Theme/Summary topic	Authors	Setting	Study design	Objective(s)	Population
Client knowledge, attitudes and believes about PrEP in pregnant or lactating people	Hamoonga et al.	Zambia	Qualitative	To explore attitudes and beliefs about PrEP among PLP	In depth interviews (IDIs) with purposively sampled 24 HIV negative pregnant and breastfeeding women (50% under 24 years)
	Young et al.	Malawi	Qualitative	To understand, from the perspective of both women and men, how male partners were involved in supporting women's oral PrEP use during pregnancy and postpartum and the impact this support had on their PrEP adherence. To understand the bidirectional impact of women's PrEP use on antiretroviral therapy (ART) use among male partners living with HIV.	IDIs with purposively recruited pregnant women and their partners (30 women and 20 men)—mix of the male population to include men living with HIV, unknown male HIV status. Women included met HIV risk indications for PrEP.
	Khumalo et al.	Eswatini	Cross sectional survey	To determine PrEP related levels of knowledge, attitudes, intentions and practices and to determine factors associated with use and intention to use PrEP	1,149 HIV negative pregnant and postpartum women
Health Care Worker (HCW) experiences and attitudes of PrEP	Pleaner et al.	South Africa	Qualitative	Understand health care worker experiences of and attitudes towards introduction of PrEP as a new HIV prevention method, and its integration within broader sexual and reproductive health (SRH) services for youth	Free text responses from 48 purposively sampled health care workers in primary health care and mobile clinics
	Wagner et al.	Kenya	Qualitative	To explore health care worker perspectives on barriers to PrEP delivery and strategies for overcoming those barriers that can be empirically tested in future studies as programs seek to integrate PrEP into existing clinical services.	Focus group discussions with 50 health care workers
	Hicks et al.	Kenya	Quantitative survey	To document available services and commodities via a modified service availability and readiness assessment (SARA) survey in PrEP experienced clinics	Health care workers with experience delivering PrEP to pregnant and postpartum women in 55 facilities
PrEP delivery	Sila et al.	Kenya	Difference-in-differences design	To test a combination of three implementation strategies (video education, HIV self-testing for repeat HIV testing, and PrEP dispensing in maternal can child health clinics) to decrease client waiting time, improve coverage of PrEP education and PrEP offer, improve PrEP knowledge, and maintain satisfaction for clients and HCWs.	960 pre-intervention (480 in comparison and 480 in intervention sites) and 959 during the intervention (478 in comparison and 481 in intervention sites)- All pregnant
	Masenyetse et al.	Lesotho	Retrospective cohort	To characterize the PrEP cascade and use patterns among pregnant and postpartum women	Routine PrEP health records of 4,098 participants in 26 health facilities—389 pregnant and postpartum women data included in analysis
	Khadka et al.	South Africa	Cohort	To examine PrEP initiation, continuation through 6 months, and persistence and evaluate the association between baseline HIV risk and PrEP delivery outcomes	486 pregnant women were included in the study, of which 16% were “adolescents” (aged 16–18 years) and 84% were “young women” (aged 19–24 years).
	Hurwitz et al.	South Africa	Single arm longitudinal	To evaluate the use of TDF/FTC as PrEP among women with potential for HIV-exposure and planning for pregnancy using group-based trajectory models	Women aged 18–35 with intention to get pregnant and partner living with HIV or of unknown HIV status (periconceptional PrEP)
PrEP Safety/Effectiveness and delivery	Scott et al.	Clinical trial data from Kenya Uganda and USA	Pharmacokinetic study	To evaluate upward-adjustment of tenofovir disoproxil fumarate (TDF)/emtricitabine (FTC) PrEP dosing during pregnancy	Modeling study
	Zewdie et al.	Uganda	Cohort	To evaluate the impact of TDF-based PrEP use on bone mineral density loss during pregnancy and investigate the effect of pregnancy on daily oral PrEP adherence and continuation.	499 HIV negative women aged 16–25 including pregnant women
	Fairlie et al.	LMICs	Review/Commentary	Review safety profiles of currently available PrEP candidates in women of child-bearing potential, pregnancy and breastfeeding and discuss pragmatic approaches for such surveillance in HIV-endemic LMICs.	Review study

Firstly, community PrEP education is critical to reducing stigma and increasing support for PrEP. Three studies explored PrEP knowledge, attitudes, and beliefs among pregnant and postpartum women and their partners. PrEP was viewed as safe and effective; however, Hamoonga et al. highlighted important concerns about side effects and potential negative impact on pregnancy and infant health. Fear of stigma was an important determinant of effective PrEP use with women without HIV including concern that partners or the community may perceive women as living with HIV or having multiple sex partners. In Eswatini (Khumalo et al.), PrEP awareness was high but accurate PrEP knowledge was incomplete. Young et al. identified PrEP misconceptions among clients who reported that PrEP improved health and could be used to treat sexually transmitted infections. Partner support was identified across several studies as a key determinant of PrEP uptake and continuation. Having a partner living with HIV was a major reason for initiating PrEP and was associated with higher adherence to both PrEP and ART. Routine data from Lesotho (Masenyetse et al.) identified a 2-fold higher follow-up among PrEP users in relationships where one partner was living with HIV. Similarly, having multiple sex partners was a common reason for PrEP use and a determinant of PrEP continuation.

Secondly, we learned about barriers and facilitators of the PrEP care continuum. Previous work has highlighted significant challenges with PrEP adherence which is critical for efficacy (11). Khadka et al. found that over 80% of pregnant adolescent girls and young women initiated PrEP in the first antenatal care visit. However, PrEP continuation reduced significantly with time and was <40% at 6-months despite the high prevalence of STIs. Similarly, Masenyetse et al. found that 40% of PrEP initiators in routine care among pregnant and postpartum had no follow-up visit, signally that barriers to PrEP continuation persist. Hurwitz et al. estimated overall PrEP adherence at 63% and identified several patterns of PrEP adherence during periconception among HIV-exposed South African women. Changes in perceived HIV risk over time may impact PrEP adherence; however, the large drop-offs and poorer PrEP persistence among women who become pregnant while on PrEP are concerning.

Thirdly, we derive insights from healthcare providers' experiences in delivering PrEP. Among providers who had no training or experience delivering PrEP (Pleaner et al.), there were significant concerns about burdening already busy clinics and the impact on other service delivery. However, in Kenya (Wagner et al.), among providers with experience delivering PrEP, delivery was viewed more favorably, as adaptable and meeting patient needs. However, PrEP delivery required provider training, was more complex compared to other services and required additional resources (Hicks et al.). Additionally, daily dosing for PrEP requiring frequent refills and access to services (e.g., long distances to clinics and waiting time) were important barriers (Hamoonga et al.). Sila et al. found that an intervention package including video education, HIV self-testing, and PrEP dispensing delivered at maternal and child health clinics significantly increased the proportion of clients counselled about PrEP and client satisfaction but was associated with increased waiting time. These findings demonstrate the need for continued research to optimize PrEP delivery.

Finally, this collection addresses PrEP effectiveness and safety in pregnancy. Fairlie et al. reviewed data on the safety profiles of available PrEP candidates including oral TDF-containing regimens, long-acting cabotegravir and the dapivirine ring. Except TDF-containing regimens, safety data on other PrEP agents is very limited in pregnancy and postpartum. They also reviewed existing drug surveillance systems in high- and low-income settings and suggested that PrEP surveillance be integrated into multiple surveillance systems. While the cost of building such systems is high, they argue that the extent of PrEP use warrants investment. Scott et al., found an increase in tenofovir/emtricitabine drug clearance throughout pregnancy, suggesting current dosing schedules may be inadequate to provide protective drug levels. Zewdie et al. found significant bone mineral density loss among pregnant women using oral TDF-based PrEP, which was likely attributed to pregnancy and not PrEP. This study was limited by small numbers of pregnant women not exposed to PrEP. Additional research is needed for robust comparisons between PrEP-exposed and unexposed populations.

In conclusion, this collection highlights important gaps in PrEP delivery among pregnant and lactating people. Ongoing discovery research will likely address pharmacokinetics and expand PrEP options; however, understanding how to scale-up PrEP delivery will require continued evaluation and adaptation to meet the needs of pregnant and postpartum women and in different regions.
